# The bacterial pathogen *Listeria monocytogenes* and the interferon family: type I, type II and type III interferons

**DOI:** 10.3389/fcimb.2014.00050

**Published:** 2014-04-28

**Authors:** Olivier Dussurget, Hélène Bierne, Pascale Cossart

**Affiliations:** ^1^Unité des Interactions Bactéries-Cellules, Institut PasteurParis, France; ^2^Inserm, U604Paris, France; ^3^INRA, USC2020Paris, France; ^4^University of Paris Diderot, Sorbonne Paris CitéParis, France

**Keywords:** listeriosis, innate immunity, immune escape, PgdA, LntA, BAHD1

## Abstract

Interferons (IFNs) are secreted proteins of the cytokine family that regulate innate and adaptive immune responses to infection. Although the importance of IFNs in the antiviral response has long been appreciated, their role in bacterial infections is more complex and is currently a major focus of investigation. This review summarizes our current knowledge of the role of these cytokines in host defense against the bacterial pathogen *Listeria monocytogenes* and highlights recent discoveries on the molecular mechanisms evolved by this intracellular bacterium to subvert IFN responses.

## Introduction

*Listeria monocytogenes* is a pathogenic Gram-positive bacillus responsible for a foodborne disease in humans and animals called listeriosis (Vázquez-Boland et al., 2001). This highly versatile bacterium can be isolated from multiple sources such as human and animal feces, soil, water, plants and food. As a common contaminant of fruits, vegetables, seafood, meat and cheese, it represents a major economic problem for the food industry. Infection usually originates from ingestion of contaminated food (Schlech et al., 1983) and may cause febrile gastroenteritis in otherwise healthy persons (Ooi and Lorber, 2005). In contrast, in immunocompromised individuals it leads to a severe invasive disease, which manifests itself as septicemia, meningitis and encephalitis. In the specific case of pregnant women, infection may cause fetal loss or neonatal bacteremia and meningitis. In the United States, incidence of listeriosis ranged from 2.5 to 3.2 cases per million population between 2004 and 2009 (Cartwright et al., 2013). In France, incidence was 3.9 per million between 2001 and 2008, and increased risk of listeriosis was noticed in people with underlying diseases, such as chronic lymphocytic leukemia (Goulet et al., 2012). While relatively rare, listeriosis is among the most deadly foodborne diseases with mortality rates reaching up to 50% depending on the clinical manifestations (Lorber, 1997). In addition to the immunological status of the patient, the clinical outcome of the disease depends on the pathogenic potential of the infecting bacteria. *L. monocytogenes* strains of serovars 1/2a, 1/2b, and 4b account for 95% of human cases and serovar 4b alone is associated to most outbreaks (Swaminathan and Gernersmidt, 2007).

The capacity of *L. monocytogenes* to survive and multiply within the gastrointestinal tract is critical for the initial infection, persistence and transmission. *L. monocytogenes* is well adapted to this environment and produces multiple factors to compete with microbiota and counteract antimicrobial peptides, acidity, hyperosmolarity, hypoxia, bile and iron deprivation (Gahan and Hill, 2005). Crossing of the intestinal epithelium is thought to occur by invasion of enterocytes, in particular goblet cells and M cells of the Peyer's patches. Invasion of enterocytes requires the specific interaction between the *Listeria* surface protein InlA and its cellular receptor E-cadherin (Lecuit et al., 2001; Disson et al., 2008), which can take place at sites of cell extrusion at the tip and other locations of intestinal villi (Pentecost et al., 2006; Nikitas et al., 2011). Indeed, as recently shown, upon interaction with E-cadherin, *Listeria* preferentially crosses the intestinal barrier by transcytosis through goblet cells (Nikitas et al., 2011). Entry through ileal Peyer's patches via M cells does not rely on InlA. It has been reported to require *Listeria* invasion protein InlB (Chiba et al., 2011). After translocation, bacteria reach lymph nodes, the liver and spleen and finally secondary target sites of infection, including the central nervous system and the placenta.

A remarkable feature of *L. monocytogenes* is its capacity to invade non-professional phagocytic cells such as enterocytes, hepatocytes and trophoblast cells. The exceptional repertoire of virulence factors necessary for entry, survival and multiplication has been extensively studied (Camejo et al., 2011; Cossart, 2011). Expression of many virulence genes relies on the transcriptional activator PrfA, whose role is pivotal for *L. monocytogenes* transition from saprophytic to intracellular lifestyle (Freitag et al., 2009; Toledo-Arana et al., 2009).

Elimination of *L. monocytogenes* is mostly based on the capacity of the host to mount an efficient cellular immune response to infection (Mackaness, 1962; Shi and Pamer, 2011). In particular, the fate of infection depends on the level of macrophage activation and on *Listeria* ability to counteract bactericidal mechanisms of host cells (Shaughnessy and Swanson, 2007; Corr and O'Neill, 2009; Stavru et al., 2011). Bacterial escape from the phagosome and avoidance of autophagy for intracytosolic replication and cell-cell spread have been well characterized. They have been shown to depend on five major virulence factors: the secreted pore-forming toxin listeriolysin O (LLO) and two phospholipases C (PlcA and PlcB) for vacuolar escape, the surface protein ActA for actin-based motility and both ActA and a surface protein of the internalin family, InlK, for autophagy evasion (Cossart, 2011; Dortet et al., 2012). Other strategies of immune escape that lead to the modulation of cytokine expression occur through a variety of mechanisms. Modifications of *L. monocytogenes* peptidoglycan by the *N*-deacetylase PgdA and the *O*-acetyl transferase OatA prevent lysozyme-dependent release of microbe-associated molecular patterns (MAMPs), activation of pathogen-recognition receptors (PRRs) and subsequent production of pro-inflammatory cytokines (Boneca et al., 2007; Aubry et al., 2011; Rae et al., 2011). The toxin LLO induces dephosphorylation of histone H3 and deacetylation of histone H4, which correlate with decreased expression of pro-inflammatory genes, such as the chemokine gene *cxcl2* (Hamon et al., 2007). The secreted internalin InlC inhibits inflammation by interacting with IKK-α, a component of the IκB-kinase complex, which is essential for NF-κB activation and expression of pro-inflammatory genes (Gouin et al., 2010). Other evasion mechanisms remain to be characterized, such as the control of the expression of IL-6 by the surface internalin InlH (Personnic et al., 2010). *L. monocytogenes* also has the capacity to modulate interferon (IFN) production during infection. Type I IFN production by infected cells can be controlled by *Listeria* multidrug efflux pumps MdrM and MdrT, via the secretion of the second messenger cyclic-di-AMP (Crimmins et al., 2008; Woodward et al., 2010; Schwartz et al., 2012). Synthesis of type III IFN has also recently been shown to be tuned by *Listeria* nucleomodulin LntA (Lebreton et al., 2011). Our knowledge concerning the role of the IFN cytokine family during listeriosis has rapidly expanded in the last few years and will be the focus of this review.

## Interferons

### The IFNs family

IFNs form a family of proteins secreted by many cell types in response to infection. They were originally named for their capacity to interfere with viral proliferation (Isaacs and Lindemann, 1957). This diverse family is composed of three groups of cytokines, namely type I-, type II-, and type III-IFNs, which are important components of innate immune responses (Table 1). Type I-IFNs consist of IFN-α, IFN-β, IFN-δ, IFN-ε, IFN-ζ, IFN-κ, IFN-ν, IFN-τ, and IFN-ω (Levy et al., 2011). Type II-IFN is composed of a single cytokine, IFN-γ (Pestka et al., 2004). Type III-IFNs are IFN-λ 1, IFN-λ 2, and IFN-λ 3 (formerly IL-29, IL-28A, and IL-28B) and IFN-λ 4 (Kotenko, 2011; Prokunina-Olsson et al., 2013). Type I- and type III-IFNs have similar signal transduction systems (see below) and are phylogenetically closer from each other than type II-IFN (Pestka et al., 2004).

**Table 1:** **The interferon family**.

**Type**	ST^*^	Human^**^	Mouse^**^	**Source**	**Target**	**Receptor**	**Signaling**
I	α	189aa-G^***^	189aa-G^***^	Ubiquitous	Ubiquitous	IFNAR1	JAK1
	β	187aa-GP	182aa-GP			IFNAR2	TYK2
	δ	–	–				STAT1
	ε	208aa	192aa				STAT2
	ζ	–	182aa-G				
	κ	207aa	199aa				
	ν	Pseudogene	–				
	τ	–	–				
	ω	195aa-G	–				
II	γ	166aa-G	155aa-G	NK, NKT, T	Ubiquitous	IFNγR1	JAK1
				APC, B		IFNγR2	JAK2
							STAT1
III	λ 1	200aa-G	Pseudogene	Ubiquitous	Epithelial cells,	IFNLR1	JAK1
	λ 2	200aa	193aa-G		Hepatocytes	IL10R2	TYK2
	λ 3	196aa	193aa				STAT1
	λ 4	179aa	Pseudogene				STAT2

* Subtypes.

** Protein length in amino-acids and protein modifications (G: glycosylation, P: phosphorylation).

*** 13 genes and a pseudogene in the human genome, 14 genes and three pseudogenes in the mouse genome.

Sequence conservation and chromosome location suggest that type I-IFN genes evolved from a single ancestor through duplication. However, the extent of type I-IFN gene diversification varies greatly depending on the species (Pestka et al., 2004). Generally, a single gene encodes the type I-IFN in fish. In contrast, multiple gene duplications and diversification led to the emergence of sub-types of type I-IFNs in mammals (Table 1). Gene duplication varies also within each sub-type. A single IFN-β gene is found in the human and mouse genomes (Decker et al., 2005; Honda et al., 2006; Durbin et al., 2013). In contrast 13 IFN-α genes and one pseudogene and 14 IFN-α genes and three pseudogenes are found in the human and mouse genomes, respectively (van Pesch et al., 2004; Durbin et al., 2013). A single gene encodes type II-IFN and four genes encode type III-IFNs in human (Decker et al., 2005; Levy et al., 2011). Of note, IFN-λ1 and IFN-λ4 are pseudogenes in mice, which prevents the study of these cytokines in this animal model (Table 1) (Fox et al., 2009).

### IFN activities: general overview

IFNs are important components of the immune system, which generally trigger cellular protective defenses in response to infection or tumor formation. Type-II IFN (IFN-γ) is a paradigm for this, being an important mediator of innate and adaptive immune responses with a key role in clearance of viral and bacterial pathogens and in tumor control. IFN-γ was first described as an antiviral protein (Wheelock and Sibley, 1965), but is now known to exhibit broader biological activities, non-redundant with that of other types of IFNs. The crucial role of IFN-γ in immunity to infection is reflected by the phenotype of mice lacking the IFN-γ receptor or the IFN-γ gene, which are highly susceptible to *Mycobacterium bovis* BCG infection (Dalton et al., 1993; Kamijo et al., 1993). Genetic deficiencies resulting in the loss of IFN-γ production or signaling in mice lead to increased susceptibility to infections by other intracellular pathogens, such as *L. monocytogenes* (see below), *Salmonella typhimurium* and some viruses (Huang et al., 1993; Harty and Bevan, 1995; Jouanguy et al., 1999). These defects also lead to the loss of tumor control (Kaplan et al., 1998). Patients with deficiencies in the IFN-γ pathway, for instance by mutation in the gene for the IFN-γ receptor 1, are characterized by severe infections with viruses and intracellular bacteria including *L. monocytogenes, Salmonella* sp. and mycobacteria (Jouanguy et al., 1996; Newport et al., 1996; Roesler et al., 1999; van de Vosse et al., 2009). IFN-γ mediates macrophage activation, i.e., increased phagocytosis and production of pro-inflammatory cytokines, of microbicidal reactive oxygen and nitrogen species, leading to clearance of intracellular pathogens (Schoenborn and Wilson, 2007). In addition, IFN-γ controls differentiation of T cells in Th1 effector cells, antigen processing and presentation by antigen-presenting cells, which participate to cellular immunity against intracellular pathogens (Schroder, 2003; Hu and Ivashkiv, 2009). The immunostimulatory and immunomodulatory properties of IFN-γ have therapeutic implications. Indeed, IFN-γ is used in patients with chronic granulomatous disease to reduce infection and mortality, although the clinical benefit has not been demonstrated in all studies (Holland, 2010).

Type I-IFNs are produced in responses to viruses, many bacteria and parasites. However, in contrast to type II IFNs, these cytokines are not always protective against bacterial infections. Indeed, the role of type I-IFNs in response to bacterial infection is complex and depends on the microorganism (Decker et al., 2005; Monroe et al., 2010; Carrero, 2013). They contribute to resistance of the host against infection by extracellular bacteria, such as *Escherichia coli*, *Helicobacter pylori*, *Streptococcus agalactiae* and *S. pneumoniae* (Mancuso et al., 2007; Watanabe et al., 2010). In contrast, they are associated with suppression of innate immune responses and increased susceptibility of the host to infection by *L. monocytogenes* (see below), *Brucella abortus*, *Chlamydia muridarum*, *Francisella novicida*, *Salmonella enterica, Staphylococcus aureus*, and *Yersinia pestis* (Auerbuch et al., 2004; Carrero et al., 2004; O'Connell et al., 2004; Qiu et al., 2008; Martin et al., 2009; Henry et al., 2010; de Almeida et al., 2011; Patel et al., 2012; Robinson et al., 2012; Archer et al., 2014). These different effects on infection are likely linked to the wide range of cellular responses induced by their downstream effectors, the products of IFN-stimulated genes (ISGs) (Schoggins et al., 2011). Although Type I-IFNs have long been known to induce antiviral response in the infected host (Isaacs and Lindemann, 1957), they can also induce apoptosis, autophagy, differentiation and migration, inhibit proliferation as well as angiogenesis and mediate cellular damage, inflammation or autoimmunity (Trinchieri, 2010). As a result, type I-IFNs have a therapeutic potential that can be used to treat tumors and viral infections (Pestka, 2007; Heim, 2013; Wilson and Brooks, 2013), while being detrimental for the host in response to a subset of pathogens.

Type III-IFNs have been discovered in 2003 (Kotenko et al., 2003; Sheppard et al., 2003) and their activities have been less extensively characterized than those of type I- and type II-IFNs. However, several studies suggest that type I and type III-IFNs share common biological activities (Levy et al., 2011; Zheng et al., 2013). Although type III-IFNs respond to different stimuli, use different receptors and are not always expressed by the same cells as type I IFNs (see below), engagement of type I- and type III-IFN receptors leads to similar transcriptional responses. Like type I-IFNs, type III-IFNs have been involved in antiproliferative and antiviral responses (Iversen and Paludan, 2010; Mordstein et al., 2010; Durbin et al., 2013; Hamming et al., 2013). Recently, type III-IFNs have been shown to be induced in response to bacterial pathogens, but their downstream effects are not yet characterized (Pietilä et al., 2010; Lebreton et al., 2011; Bierne et al., 2012).

### Activation of IFNs

**Figure 1:**
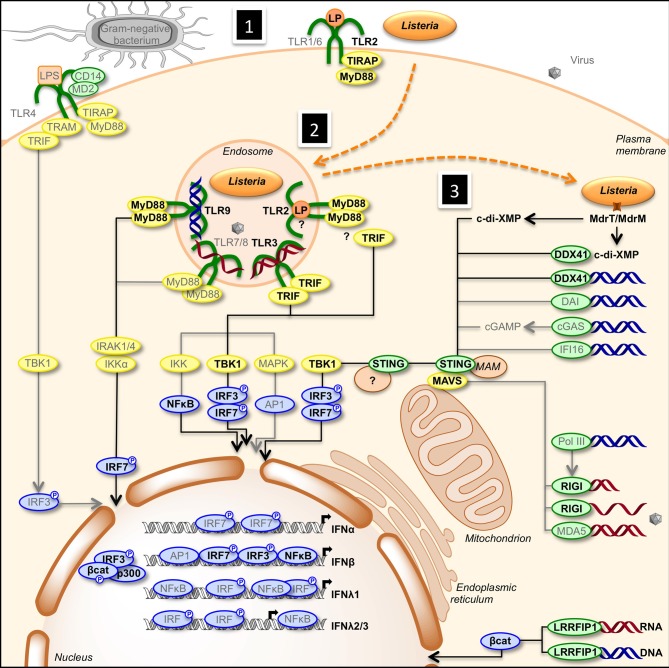
**Activation of type I- and type III-IFNs by infection**. (1) Cells detect pathogens by membrane-bound PRRs of the TLR family, which trigger the IFN response. Infection of cells with extracellular Gram-negative bacteria leads to activation of TLR4/CD14/MD2 by LPS. Following TLR4 ligation, IRF3 is activated via the adaptors TRAM and TRIF, and TBK1. (2) Intracellular bacteria and viruses are recognized by TLRs located in the endosome. TLR2, TLR3, TLR7/8, and TLR9 detect lipoproteins, dsRNA, ssRNA, and unmethylated CpG DNA, respectively. Engagement of the receptors leads to the induction of MyD88- and TRIF-dependent pathways involving IKK-, TBK1-, and MAPK-mediated activation of NF-κ B, IRF3, IRF7, and AP1, ultimately resulting in transcriptional activation of IFN genes. (3) Infection of cells leads to the release of cyclic-di-nucleotides (c-di-XMP) and nucleic acids (RNA in red, DNA in blue) in the cytosol that signal the presence of microbes and induce expression of interferons. Cyclic-di-nucleotides secreted by bacteria, e.g., through multidrug resistance MdrT and MdrM in *Listeria*, can activate the protein STING directly and via the cytosolic sensor DDX41. Cytosolic DNA is recognized by DDX41, DAI, cGAS, and IFI16. cGAS converts dsDNA to cGAMP, which stimulates STING. RNA polymerase III (PolIII) can convert cytosolic AT-rich dsDNA to uncapped 5′-triphosphate RNA that, as ssRNA, is sensed by RIGI. Another cytosolic RNA sensor, MDA5, and RIGI signal via the mitochondrial adaptor MAVS and STING. Activated STING recruits TBK1, which phosphorylates transcription factors IRF3 and IRF7. Upon activation, IRFs translocate to the nucleus where they bind to the promoter of type I IFN genes. Bacterial RNA and DNA additionally activate the cytosolic sensor LRRFIP1 that stimulates β-catenin phosphorylation and nuclear translocation where it binds to IRF3, recruits the histone acetyltransferase p300 to the enhanceosome leading to IFNβ transcription. Gray font and arrows: general infection pathways, bold black font and arrows: *Listeria* infection pathways. Red strands, RNA; blue strands, DNA. AP1, activator protein-1; CD14, cluster of differentiation 14; c-di-XMP, cyclic-di-nucleotide; cGAMP, cyclic-GMP-AMP; cGAS, cGAMP synthase; DAI, DNA-dependent activator of IFN-regulatory factors; DDX41, DEAD (aspartate-glutamate-alanine-aspartate)-box polypeptide 41; ER, endoplasmic reticulum; IFI16, IFN-inducible protein 16; IKK, Iκ B kinases; IRAK, interleukin-1 receptor-associated kinase; LP, lipoprotein; LPS, lipopolysaccharide; LRRFIP1, leucine-rich repeat flightless-interacting protein 1; MAM, mitochondria associated ER membranes; MAPK, mitogen-activated protein kinase; MAVS, mitochondrial antiviral signaling; MD2, myeloid differential protein-2; MDA5, melanoma differentiation-associated gene 5; MyD88, myeloid differentiation factor 88; NF-κ B, nuclear factor of kappa light polypeptide gene enhancer in B-cells; PolIII, RNA polymerase III; RIGI, retinoic acid inducible gene I; STING, stimulator of IFN genes; TBK1, TANK-binding kinase-1; TIRAP, TIR domain-containing adapter protein; TLR, Toll-like receptor; TRAM, TRIF-related adaptor molecule; TRIF, TIR domain-containing adaptor inducing IFN-β.

Transcription of IFN genes is induced rapidly in response to microbial infection. Type I-IFNs can be produced by all cells, while type III-IFNs are secreted by specific cell types, including dendritic and epithelial cells. Type I- and type III-IFNs activation is initiated by detection of MAMPs by PRRs such as endosomal transmembrane Toll-like receptors and cytosolic receptors (Stetson and Medzhitov, 2006; Monroe et al., 2010). Upon recognition of MAMPs, PRRs trigger diverse signaling pathways that involve adaptor proteins and cytosolic or organelle-bound protein scaffolds activating kinases converging to phosphorylation of transcription factors and their subsequent translocation into the nucleus (Figure 1). IRF1, IRF3, IRF4, IRF5, IRF7, and IRF8 are important for transcription of the IFN-α genes, with IRF7 considered as the master regulator of IFN-α response (Honda et al., 2005; Tailor et al., 2007; Levy et al., 2011). Regulation of the IFN-β gene is more complex. Activated IRF3, IRF7, AP-1, and NF-κ B bind to the enhancer/promoter regions of the IFN-β gene and participate to the formation of the enhanceosome, which alters chromatin structure and allows transcription (Panne et al., 2007; Panne, 2008). In contrast, IRFs and NF-κ B independently activate transcription of type III-IFN genes (Iversen and Paludan, 2010).

Regulation of the IFN-γ gene expression is different from that of type I and type III-IFN genes. NK cells and NKT cells are effectors of the innate immune response and primary sources of IFN-γ. Mature NK and NKT cells quickly react to infection by inducing IFN-γ secretion. Upon recognition of ligands expressed on infected cells, NK cell activating-receptors trigger signaling cascades involving adaptor proteins and protein tyrosine kinases leading to activation of Ras/Sos, PLC-γ and MAPK pathways and induction of IFN-γ production (Schoenborn and Wilson, 2007). In addition to receptors, IL-2, IL-15, IL-12, IL-18, and type I-IFNs also contribute to induction of IFN-γ production by NK cells (Newman and Riley, 2007; Schoenborn and Wilson, 2007; Marçais et al., 2013). Similarly, IL-12 and IL-18 induce IFN-γ production by NKT cells (Godfrey and Berzins, 2007). In NK and NKT cells, the IFN-γ gene locus is transcriptionally permissive within accessible chromatin and allows rapid IFN-γ expression upon activation of transcription factors, such as AP-1, NF-κ B, STAT4, and T-bet (Glimcher et al., 2004; Schoenborn and Wilson, 2007; Lazarevic et al., 2013). In addition, naive CD4 and CD8 T cells can differentiate into Th1 CD4 effector T cells and CD8 cytotoxic T lymphocytes capable of IFN-γ secretion (Wilson et al., 2009). IFN-γ production by CD4 and CD8 T cells depends on IL-12, IL-18 and IFN-γ itself and share many signaling pathways with NK cells. Multiple transcription factors act at the IFN-γ promoter, e.g., AP-1, ATF-2/c-Jun, C/EBP, Eomes, Ets-1, NFAT, NF-κB, Runx3, STATs and T-bet (Schoenborn and Wilson, 2007; Samten et al., 2008; Wilson et al., 2009; Lazarevic et al., 2013). Moreover, distal regulatory elements modify the chromatin and remodel the IFN-γ gene locus to facilitate IFN-γ production (Wilson et al., 2009).

### IFN receptors and signal transduction

**Figure 2:**
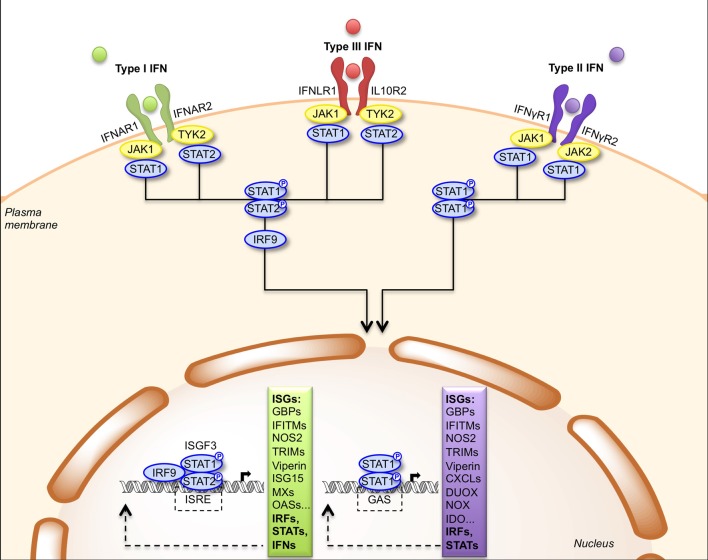
**Signal transduction by the type I, type II and type III IFN receptors**. Engagement of type I and type III IFNs by their respective receptors, IFNAR1/IFNAR2 and IFNLR1/IL10R2, triggers phosphorylation of JAK1/TYK2 kinases that activate STAT1 and STAT2. Phosphorylated STAT1/STAT2 heterodimers bind IRF9 forming the ISGF3 complex, which translocates to the nucleus where it induces expression of genes with ISRE-dependent promoters. Engagement of type II IFN by the IFNγR1/IFNγR2 receptor induces phosphorylation of JAK1/JAK2 kinases that activate STAT1. Phosphorylated STAT1 homodimers translocate to the nucleus and induces expression of genes with GAS-dependent promoters. CXCL, CXC chemokine ligand; DUOX, dual oxidase; GAS, gamma-IFN activated site; GBP, guanylate-binding protein; IDO, indoleamine 2,3-dioxygenase; IFITM, IFN-induced transmembrane protein; IRF9, IFN-regulatory factor 9; ISG, IFN-stimulated gene; ISGF3, IFN-stimulated gene factor 3; ISRE, IFN-stimulated response element; JAK, Janus activated kinase; MX, myxovirus resistance; NOS2, nitric oxide synthase 2; NOX, NADPH oxidase; OAS, oligoadenylate synthetase; STAT, signal transducer and activator of transcription; TRIM, tripartite motif; TYK2, tyrosine kinase 2.

IFNs are rapidly secreted upon infection and then bind to their receptors on the surface of target cells (Table 1). Type I-IFNs bind the ubiquitous IFNAR receptor, which consists of two chains, IFNAR1 and IFNAR2 (Piehler et al., 2012). Type III-IFNs bind and signal through a different receptor complex, made of two chains: IFNLR1 (also known as IL-28Rα) and IL10R2. This receptor is expressed primarily by epithelial cells and hepatocytes (Iversen and Paludan, 2010). Thus, the physiological roles of type I- and type III-IFNs are distinct because of the different distribution of their receptors in tissues, type III-IFNs acting predominantly at mucosal surfaces (Mordstein et al., 2010; Durbin et al., 2013). Type I- and type III-IFNs use different receptors but trigger the same JAK-STAT signal transduction cascade involving TYK2, JAK1, STAT1, and STAT2 albeit with different kinetics (Figure 2) (Marcello et al., 2006). Ultimately, STAT1, STAT2, and IRF9 form a transcription factor complex, referred to as ISGF3, which translocates to the nucleus and binds to IFN-stimulated responsive elements (ISRE) in the promoter of ISGs (Schindler et al., 2007). Type II-IFN, uses a heterodimeric receptor consisting of IFNγR1 and IFNγR2 chains, expressed by many cell types (Bach et al., 1997). IFN-γ activates JAK1, JAK2 and STAT1, leading to transcription of genes bearing a γ-activation sequence (GAS) in their promoter (Figure 2) (Schindler et al., 2007).

## IFNs and *Listeria* infection

### Induction of IFNs in response to *Listeria* infection

An initial innate immune response is triggered when *Listeria* MAMPs activate PRRs of host cells such as epithelial cells and macrophages (Figure 1). Infection induces a robust type I-IFN response. In mice, macrophages have been identified as the major source of IFN-β (Stockinger et al., 2009). *In vitro*, IFN-β production by bone-marrow-derived murine macrophages has been shown to require bacterial escape from the phagosome and activation of cytosolic surveillance pathways (O'Riordan et al., 2002). Induction of IFN-β depends on the adaptor protein STING and the cytosolic PRR DDX41, which are activated by bacterial secondary messengers c-di-AMP and c-di-GMP and by bacterial DNA (Woodward et al., 2010; Burdette et al., 2011; Sauer et al., 2011; Parvatiyar et al., 2012; Archer et al., 2014). STING is a direct receptor for cyclic-dinucleotides, including the cellular second messenger cyclic GMP-AMP (cGAMP) which is produced by the cytosolic sensor cGAMP synthase (cGAS) upon interaction with microbial DNA (Ablasser et al., 2013; Gao et al., 2013; Wu et al., 2013; Sun et al., 2013; Schoggins et al., 2014). Interestingly, type I-IFN production requires activation of the RIG-I helicase by *Listeria* RNA in non-immune cells lacking a functional STING signaling pathway (Abdullah et al., 2012; Hagmann et al., 2013). Another cytosolic PRR, the leucine-rich repeat-containing protein LRRFIP1, has also been implicated in IFN-β production by mouse primary peritoneal macrophages in response to *Listeria* infection, possibly by sensing double stranded DNA and RNA (Yang et al., 2010). While production of IFN-β in response to *Listeria* infection is independent from TLRs in bone-marrow-derived macrophages (McCaffrey et al., 2004; Stockinger et al., 2004; O'Connell et al., 2005), TLR-2 contributes significantly to IFN-β secretion by peritoneal macrophages, suggesting that specific macrophage populations have evolved different recognition strategies in response to *Listeria* infection (Aubry et al., 2012).

*Listeria* infection has recently been shown to induce type III-IFN gene expression in cells of epithelial origin, such as intestinal and trophoblast cells and hepatocytes (Lebreton et al., 2011; Bierne et al., 2012). Similar to type I-IFN, type III-IFN induction is triggered by intracellular *Listeria* (Bierne et al., 2012).

*Listeria* infection also triggers a rapid and robust IFN-γ response. After intravenous infection of mice with *L. monocytogenes*, NK and T cells are the main sources of IFN-γ (Thale and Kiderlen, 2005; Bou Ghanem et al., 2009). IFN-γ producing V1δ^+^−γδ T cells are other murine immune cells induced at an early stage of *Listeria* infection in mice inoculated intraperitoneally (Hamada et al., 2008). Using oral infection of mice, the natural route of infection in permissive hosts, *L. monocytogenes* has been shown to induce IFN-γ production by intraepithelial lymphocytes of the small intestine (Okamoto, 1994). More recently, human E-cadherin (hEcad) expressing mice, a mouse line permissive for *Listeria* oral infection (Lecuit et al., 2001), were used to study cells involved in intestinal mucosal immunity. Infection induced IFN-γ production in NK cells of the small intestine (Reynders et al., 2011).

### Role of IFNs during *Listeria* infection

The production of IFN-γ by immune cells promotes bacterial clearance and is thus critical in controlling primary *L. monocytogenes* infections (Zenewicz and Shen, 2007). Injection of neutralizing monoclonal anti-IFN-γ antibodies in mice infected intraperitoneally with *L. monocytogenes* inhibits macrophage activation and increases the mortality rate (Buchmeier and Schreiber, 1985). In addition, resistance of IFN-γ gene or IFN-γ receptor knock-out mice infected intravenously with *L. monocytogenes* is severely impaired (Huang et al., 1993; Harty and Bevan, 1995). Recent work using cell-type specific inactivation of STAT1 in mice elegantly demonstrated the key role of IFN-γ and STAT1 in macrophage activation and clearance of *Listeria* (Kernbauer et al., 2012). Interestingly, the role of STAT1 was extremely different after infection of immunized mice. STAT1 signaling in T cells and dendritic cells was critical for adaptive immunity to *Listeria*, while IFN-γ-activated macrophages were not essential anymore once memory cells were produced. Upon oral infection of hEcad mice with *Listeria*, IFN-γ contributes to the control of bacterial burden in the intestine and of bacterial dissemination to other organs. For instance, blocking IFN-γ with neutralizing antibodies increases *Listeria* load in the small intestine, the mesenteric lymph nodes and in the spleen of mice infected orally (Reynders et al., 2011).

In contrast to IFN-γ, type I-IFN is beneficial to *L. monocytogenes*. Mice lacking type I-IFN receptor or IRF3 are more resistant to *Listeria* intraperitoneal or intravenous infection (Auerbuch et al., 2004; Carrero et al., 2004; O'Connell et al., 2004; Garifulin et al., 2007; Jia et al., 2009). The role of type I-IFNs in increasing host susceptibility could be explained by modulation of components of the immune response involved in controlling bacterial growth such as induction of T cell apoptosis, resulting in greater IL-10 secretion by phagocytic cells, in turn dampening the innate immune response (Carrero and Unanue, 2006), the downregulation of IFN-γR (Rayamajhi et al., 2010; Kearney et al., 2013), or neutrophil recruitment (Brzoza-Lewis et al., 2012). As shown recently, STING-dependent activation of type I-IFN reduces the adaptive immune response to *L. monocytogenes* (Archer et al., 2014). In contrast, recent studies showed that type I-IFNs can also play a beneficial role for the host during *Listeria* infection, pointing to the infection route and the timing of type I-IFN production as determinative factors (Pontiroli et al., 2012; Kernbauer et al., 2013).

Interestingly, different strains of *L. monocytogenes* have been shown to vary greatly in their capacity to induce IFN-β (Reutterer et al., 2008; Schwartz et al., 2012). The LO28 strain hyperinduces IFN-β (Reutterer et al., 2008). This strain bears a non-functional BrtA (also named TetR), the transcriptional repressor of the multidrug efflux pump MdrT (Schwartz et al., 2012; Yamamoto et al., 2012). In *Listeria*, MdrT allows secretion of c-di-AMP, which triggers IFN-β. Thus, derepression of MdrT in the LO28 strain promotes IFN-β production. Of note, high expression of MdrT in LO28 correlates with both induction of IFN-β and lower virulence. Another *Listeria* multidrug resistance transporter, MdrM, has been involved in the stimulation of IFN-β production, possibly by secreting c-di-AMP (Crimmins et al., 2008; Woodward et al., 2010; Witte et al., 2012).

The role of type III-IFNs during listeriosis remains to be determined. Since *Listeria* colonizes several tissues of epithelial origins, such as the liver, intestine and placenta, it is tempting to speculate that IFN-λs play a role in the interaction of *Listeria* with epithelia. However, a prerequisite to address this question is the establishment of a new animal model, i.e., a mouse line expressing a human E-cadherin, thus permissive for *Listeria* infection of epithelia (Lecuit et al., 2001) and impaired in type III-IFN responses, such as IL28Rα knockout mice (Mordstein et al., 2010). One should keep in mind that the mouse model is not optimal to address the role of type III-IFN in human listeriosis. Indeed, *IFN*-λ*1* is a pseudogene in mice, while human cells produce this cytokine upon infection with *L. monocytogenes*. In addition, the type III-IFN receptor is expressed at very low levels in the mouse liver and the *IFN*-λ response of the mouse liver is very weak (Mordstein et al., 2010). In line with this, it has been recently shown that mouse hepatocytes, in contrast to human hepatocytes, are not responsive to *IFN*-λ (Hermant et al., 2014).

### Role of IFN-stimulated genes during *Listeria* infection

The beneficial or detrimental effects of IFNs on *Listeria* infection rely on the functional properties of their downstream effectors. Indeed, IFNs elicit expression of hundreds of interferon-stimulated genes (ISGs), which encode proteins involved in a broad range of cellular functions (reviewed in MacMicking, 2004). However, while about 2,000 ISGs have been identified so far (Rusinova et al., 2012), their functions in immunomodulation remain to be characterized. To date, the contribution of interferon-induced proteins on *Listeria* infection has mostly been studied in the context of the IFN-γ pathway.

The antilisterial activity of IFN-γ in phagocytic cells involves induction of oxidative and nitrosative defences, via increased expression of enzymes that control production of reactive oxygen and nitrogen species, such as NOX2/CYBB, DUOX2, and iNOS/NOS2 (MacMicking, 2012). These enzymes play an important role in protecting infected cells against *Listeria* cytoinvasion (Myers et al., 2003; Lipinski et al., 2009). The assembly of these enzymes requires IFN-γ–inducible guanosine triphosphatases (GTPases) of the Gbp (guanylate binding protein) family (Boehm et al., 1998), which not only participate to oxidative pathways but also regulate autophagy (Kim et al., 2011). Several Gbps have been shown to protect cells from *Listeria* infection by coordinating a potent oxidative and vesicular trafficking program (Kim et al., 2011). IFN-γ also induces the expression of many nuclear genes encoding mitochondrial respiratory chain machinery, via activation of the nuclear receptor ERRα (estrogen-related receptor α). ERRα contributes to mitochondrial ROS production and efficient clearance of *L. monocytogenes* (Sonoda et al., 2007). A family of IFN-γ-induced chemokines (CXCL9, CXCL10, CXCL11) displays direct antimicrobial activity against *L. monocytogenes* (Cole et al., 2001). In dendritic cells, one of the IFN-γ-associated ISGs is the immunoregulatory enzyme indoleamine 2,3-dioxygenase (IDO), a key enzyme of the tryptophan metabolism. IDO is proposed to play a role in the containment of *Listeria* within granulomatous structures, thus avoiding massive T cell activation (Popov et al., 2006).

The function of type I IFN-associated ISGs in *Listeria* infection is less documented. Zwaferink et al. have observed that upregulation of iNOS/NOS2 by IFN-β promotes necrotic death of macrophages (Zwaferink et al., 2007). Additionally, several interferon-inducible proteins belong to inflammasomes; thus, type I IFN may potentiate inflammasome activation and cell death by pyroptosis (Malireddi and Kanneganti, 2013). Yet, the link between these effectors and the observed harmful effects of type I IFNs on the host is still unclear. Likewise, the role of effectors induced by type III IFNs in *Listeria* infection of epithelia is not understood.

Of interest, a subset of ISGs is amongst the most induced genes in the intestinal tissue of gnotobiotic humanized mice infected orally with *L. monocytogenes* (Archambaud et al., 2012). However, which type of IFNs triggers this response and for which function on the intestinal mucosa remain to be explored. In addition, IFN-independent pathways may contribute to expression of these ISGs.

### Subversion of IFN responses by *Listeria*

*Listeria* has evolved several mechanisms to avoid immune detection and evade IFN responses. It has been demonstrated that deacetylation of *Listeria* peptidoglycan by the deacetylase PgdA confers resistance to host lysozyme, thus preventing release of MAMPs, such as DNA, RNA and lipopeptides, that trigger IFN-β production (Boneca et al., 2007). *Listeria pgdA* mutants are rapidly killed in murine macrophages, which produce lysozyme, and induce a strong secretion of IFN-β compared to wild-type *Listeria*. The role of PgdA is not limited to the control of type I-IFN production as a *pgdA* mutant hyperinduces pro-inflammatory cytokines as well. Modification of peptidoglycan by PgdA is an extremely efficient mechanism of immune escape used by *Listeria*, which correlates with its critical role in virulence.

Remarkably, *Listeria* has evolved a sophisticated strategy to modulate, either negatively or positively, the expression of ISGs in epithelial cells, by targeting a chromatin-repressive complex, BAHD1 (Bierne et al., 2009; Lebreton et al., 2011, 2012). Indeed, *Listeria* infection promotes, albeit via an unknown mechanism, the targeting of BAHD1 at the promoter of a set of ISGs, thereby downregulating type I- and type III-IFN responses. On the other hand, *Listeria* can produce a nucleomodulin, LntA, which when secreted by intracellular bacteria, enters the nucleus of infected cells, binds BAHD1 and inhibits its function (Lebreton et al., 2012, 2014). Thus, LntA stimulates IFN responses. Consistent with the presence of HDAC1/2 in the BAHD1-associated complex, the level of acetylation of lysine 9 on histone H3, which is a mark of active chromatin, increases at the promoters of ISGs in the presence of LntA. When, in which host conditions, and how LntA targets BAHD1 specifically at ISGs remains an open question. The LntA-mediated stimulation of type III-IFN responses might support localized pro-bacterial conditions, as was proposed for IFN-I responses.

## Concluding remarks

We have an extensive knowledge of the molecular and cellular mechanisms involved in *Listeria*-host interactions. Yet, our understanding of the immune response to *Listeria*, and more specifically the role IFNs and of their downstream effectors, is far from complete and often relies on studies performed in cultured cells or in mice. However, murine and human listeriosis differ in many aspects (Lecuit, 2007; Hoelzer et al., 2012). For instance, E-cadherin, the major receptor for *Listeria* in epithelial cells, is not functional for *Listeria* uptake in the mouse. Thus, the route of entry of *Listeria* is not strictly the same in mice and humans. Moreover, ISGs induced in response to infection are not identical in mice and humans. Additionally, murine hepatocytes do not respond to type III-IFNs (Hermant et al., 2014), precluding the study of these IFNs during infection by human hepatotropic pathogens, such as *L. monocytogenes*. Altogether, species-specific differences provide limits to the use of mouse models in characterizing IFN pathways engaged during *Listeria* infection in humans, especially in key epithelial organs such as the gut, liver and placenta. It will be important to perform future studies using adapted animal models, such as humanized mice permissive to oral infection or transgenic mice with human xenografts (Walters et al., 2006), since the effect of type I-IFN on *Listeria* infection depends on the route and time of infection (Pontiroli et al., 2012; Kernbauer et al., 2013) and type III-IFN requires bacterial interaction with epithelia (Bierne et al., 2012). Finally, numerous ISGs are induced upon *Listeria* infection *in vitro*, but the relevant ISGs and their cellular functions remain to be identified. Validation of ISGs identified in cultured cells in adequate *in vivo* models or deduced from analyses of patient samples, will be required to address the complex role of IFNs and bacterial subversion strategies and provide new insights into *Listeria* pathogenesis.

### Conflict of interest statement

The authors declare that the research was conducted in the absence of any commercial or financial relationships that could be construed as a potential conflict of interest.
